# Anisotropic Panglial Coupling Reflects Tonotopic Organization in the Inferior Colliculus

**DOI:** 10.3389/fncel.2018.00431

**Published:** 2018-11-27

**Authors:** Simon L. Wadle, Vanessa Augustin, Julia Langer, Ronald Jabs, Camille Philippot, Dennis J. Weingarten, Christine R. Rose, Christian Steinhäuser, Jonathan Stephan

**Affiliations:** ^1^Animal Physiology Group, Department of Biology, University of Kaiserslautern, Kaiserslautern, Germany; ^2^Institute of Neurobiology, Heinrich Heine University Düsseldorf, Düsseldorf, Germany; ^3^Institute of Cellular Neurosciences, Medical Faculty, University of Bonn, Bonn, Germany

**Keywords:** astrocytes, oligodendrocytes, auditory brainstem, IC, gap junctions, connexin 43, connexin 30

## Abstract

Astrocytes and oligodendrocytes in different brain regions form panglial networks and the topography of such networks can correlate with neuronal topography and function. Astrocyte-oligodendrocyte networks in the lateral superior olive (LSO)—an auditory brainstem nucleus—were found to be anisotropic with a preferred orientation orthogonally to the tonotopic axis. We hypothesized that such a specialization might be present in other tonotopically organized brainstem nuclei, too. Thus, we analyzed gap junctional coupling in the center of the inferior colliculus (IC)—another nucleus of the auditory brainstem that exhibits tonotopic organization. In acute brainstem slices obtained from mice, IC networks were traced employing whole-cell patch-clamp recordings of single sulforhodamine (SR) 101-identified astrocytes and concomitant intracellular loading of the gap junction-permeable tracer neurobiotin. The majority of dye-coupled networks exhibited an oval topography, which was preferentially oriented orthogonal to the tonotopic axis. Astrocyte processes showed preferentially the same orientation indicating a correlation between astrocyte and network topography. In addition to SR101-positive astrocytes, IC networks contained oligodendrocytes. Using Na^+^ imaging, we analyzed the capability of IC networks to redistribute small ions. Na^+^ bi-directionally diffused between SR101-positive astrocytes and SR101-negative cells—presumably oligodendrocytes—showing the functionality of IC networks. Taken together, our results demonstrate that IC astrocytes and IC oligodendrocytes form functional anisotropic panglial networks that are preferentially oriented orthogonal to the tonotopic axis. Thus, our data indicate that the topographic specialization of glial networks seen in IC and LSO might be a general feature of tonotopically organized auditory brainstem nuclei.

## Introduction

Astrocytes are coupled by gap junctions and thereby form large networks that serve, e.g., ion and neurotransmitter homeostasis (Wallraff et al., 2006; Pannasch et al., 2011; Chaturvedi et al., 2014; Rose and Chatton, 2016). Gap junctional coupling also includes other macroglial cells, but the degree of panglial coupling is heterogeneous among different brain regions: astrocytes and oligodendrocytes form panglial networks, e.g., in cortex, hippocampus, thalamus, lateral superior olive (LSO), and corpus callosum (Maglione et al., 2010; Griemsmann et al., 2015; Augustin et al., 2016; Moshrefi-Ravasdjani et al., 2017; Claus et al., 2018). Interestingly, in the corpus callosum, glial networks additionally contain NG2 glia (Maglione et al., 2010; Moshrefi-Ravasdjani et al., 2017), which is not observed in other brain areas (Wallraff et al., 2004; Houades et al., 2008; Muller et al., 2009; Xu et al., 2014; Griemsmann et al., 2015).

Radial diffusion of gap junction tracers typically gives rise to spherical, tracer-filled networks in many brain regions (Binmoller and Muller, 1992; Houades et al., 2006). However, some regions also show anisotropic networks. Such anisotropy is present predominantly, but not exclusively, in sensory systems, which exhibit a strong correlation between anatomical and functional organization (Houades et al., 2008; Roux et al., 2011; Augustin et al., 2016; Claus et al., 2018; Condamine et al., 2018). The anisotropy of tracer spreading is promoted by astrocyte anisotropy (Anders et al., 2014; Augustin et al., 2016; Ghezali et al., 2018): astrocytes with processes occupying an oval territory give rise to an oval network of tracer-filled cells.

**FIGURE 1:**
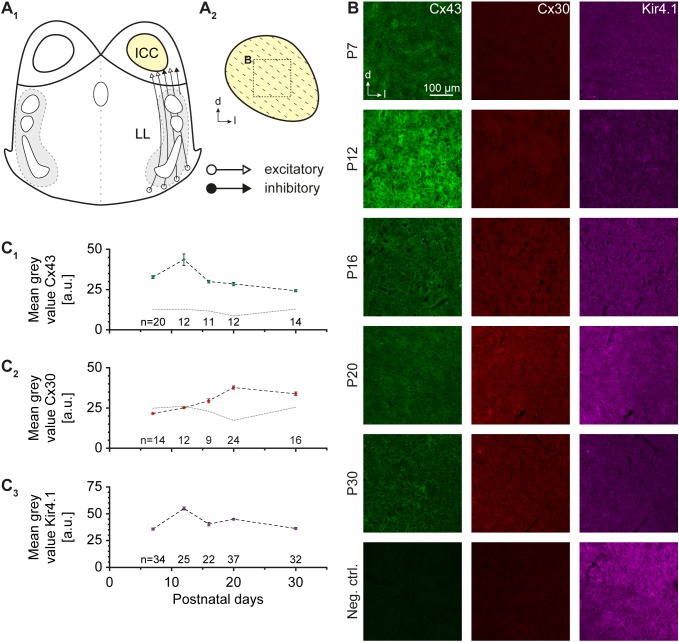
Cx43, Cx30, and Kir4.1 immunoreactivity. **(A)** Schematic drawing of an auditory brainstem slice containing the central part of the inferior colliculus (ICC; **A_1_**). The tonotopic organization is indicated by dotted lines **(A_2_)**. The dotted box denotes the area in which Cx expression was analyzed. **(B)** Immunoreactivity for Cx43 was maximal at P12 and decayed with age, whereas Cx30 levels increased at later developmental stages. Kir4.1 exhibited a moderately elevated level during the second postnatal week. The last row depicts negative controls at P20, in which the primary antibodies for Cx43 and Cx30 were omitted. **(C)** Developmental profile of Cx43 **(C_1_)**, Cx30 **(C_2_)**, and Kir4.1 expression **(C_3_)**. The dotted gray lines in **(C_1,2_)** denote developmental background fluorescence signals at P7–30 obtained after omitting primary antibodies. *n* represents the number of analyzed slices and is provided within the diagrams; shown are mean values ± SEM.

In the LSO, most astrocyte-derived tracer-filled networks exhibit an oval shape that is oriented orthogonally to the tonotopic axis (Augustin et al., 2016). This orientation correlates with dendrite topography and isofrequency bands (Sanes et al., 1992a,b; Rietzel and Friauf, 1998; Kandler et al., 2009) and suggests a reduced crosstalk by the glial network due to putative anisotropic redistribution of signaling elements (cf. Augustin et al., 2016). We hypothesized that this specialization of glial networks is not a unique feature of the LSO, but might be present in further tonotopically organized auditory brainstem nuclei as well. The inferior colliculus (IC) is the most rostral auditory brainstem center. Like the LSO, the IC is tonotopically organized (see Figure 1; Merzenich and Reid, 1974; Huang and Fex, 1986; Ball et al., 2007; Cruz et al., 2007) and IC neurons possess dendrites with narrowed topography that are oriented orthogonal to the tonotopic axis (Oliver and Morest, 1984; Bal et al., 2002; Malmierca et al., 2011; Ghirardini et al., 2018). Astrocytes are homogeneously distributed within the central IC and form gap junction networks (Hafidi and Galifianakis, 2003; Ball et al., 2007; Gandhi et al., 2010; Ghirardini et al., 2018). Most cells within IC coupling networks were found to be immunopositive for the calcium-binding protein S100β that is expressed by many astrocytes in various brain regions. In turn, the number of oligodendrocytes within the networks was considered to be low (Ball et al., 2007). So far, it was not explicitly addressed whether the tonotopic organization of the IC is reflected by glia. Although there is some information on the distribution and properties of macroglial cells in this particular brain region, the organization of astrocyte morphology and the gap junctional coupling was not characterized in detail, yet.

Here, we addressed this question by analyzing the organization of gap junctional coupling in IC during early postnatal development in the mouse brain. We found evidence for elevated expression of connexin (Cx) 43 during early postnatal stages, whereas Cx30 expression was detected at first during the third postnatal week. Sulforhodamine (SR) 101-labeled IC astrocytes gave rise to large, anisotropic tracer-labeled networks that were oriented predominantly orthogonal to the tonotopic axis. This orientation correlated with the topography of astrocyte processes and dendrites of IC neurons. In addition, these networks contained oligodendrocytes with an astrocyte:oligodendrocyte (A:O) ratio of about 3:1. Finally, panglial IC networks were able to efficiently redistribute locally elevated Na^+^ indicating functional coupling through rapid exchange of ions. Taken together, our data show that astrocytes and gap junction coupling in the IC are specialized to follow the tonotopic organization principle of the nucleus.

## Materials and Methods

### Immunohistochemistry

Experiments were performed on wild type C57BL/6 mice of both genders in accordance with the German Animal Protection Law (TSchG) as well as guidelines for the welfare of laboratory animals released by the European Community Council Directive. They also followed the NIH guidelines for the care and use of laboratory animals. In accordance with TSchG (section 4, paragraph 3), no additional approval for *post mortem* removal of brain tissue was necessary. All chemicals were purchased from Sigma-Aldrich or AppliChem, if not stated differently. For immunohistochemistry, mice at P7, P12, P16, P20, and P30 were used. The animal perfusion and tissue preparation was done as described earlier (Hirtz et al., 2011) and the tissue was subsequently processed for Cx43, Cx30, and Kir4.1 antibody labeling as described before (Augustin et al., 2016). Primary antibodies (rabbit anti-connexin 43, C6219, Sigma-Aldrich; rabbit anti-connexin 30, 700258, Invitrogen; mouse anti-Kir4.1, H00003766-M01, Novus Biologicals) were diluted 1:500. Secondary antibodies (alexa fluor 488 goat anti-rabbit, A-11034, Invitrogen; alexa fluor 568 goat anti-mouse, A-11031, Invitrogen) were diluted 1:1,000. For background correction of signal intensities, negative controls were performed. Background signal intensities were independent of animal age; for Cx43 and Cx30, we yielded at P7–30 a background mean gray value of 11.8 ± 0.7 a.u. (*n* = 6) and 23.4 ± 1.3 a.u. (*n* = 6; Figure 1B, bottom row), respectively. Both are indicated in the diagrams (Figure 1C_1,2_).

### Preparation of Acute Tissue Slices

Acute coronal brainstem slices were prepared as described earlier (Ghirardini et al., 2018). Slices were obtained from wild type C57BL/6 and PLP-GFP mice (Fuss et al., 2000)—animal age was P10–12 and P12-13, respectively. After decapitation, brains were quickly dissected and transferred into ice-cold solution, in which 270-μm-thick slices were cut using a vibratome (VT1200 S, Leica; HM650V, Microtome, Microm International GmbH). The cutting saline contained (in mM): 26 NaHCO_3_, 1.25 NaH_2_PO_4_, 2.5 KCl, 1 MgCl_2_, 2 CaCl_2_, 260 D-glucose, 2 Na-pyruvate, and 3 myo-inositol, pH 7.4, bubbled with carbogen (95% O_2_, 5% CO_2_). After sectioning, slices were transferred to artificial cerebrospinal fluid (ACSF) containing (in mM): 125 NaCl, 25 NaHCO_3_, 1.25 NaH_2_PO_4_, 2.5 KCl, 1 MgCl_2_, 2 CaCl_2_, 10 D-glucose, 2 Na-pyruvate, 3 myo-inositol, and 0.44 ascorbic acid, pH 7.4, bubbled with carbogen. Slices were incubated for 30 min at 37°C in 0.5-1 μM SR101 and washed for another 30 min at 37°C in SR101-free ACSF (Stephan and Friauf, 2014; Ghirardini et al., 2018).

### Electrophysiology

Whole-cell patch-clamp experiments were performed at room temperature at an upright microscope equipped with infrared differential interference contrast (Eclipse FN1, Nikon, 60 × water immersion objective, N.A. 1.0) and an infrared video camera (XC-ST70CE, Hamamatsu) using either a double patch-clamp EPC10 or EPC7 amplifier and “PatchMaster” or TIDA software (HEKA Elektronik). The pipette solution contained (in mM): 140 K-gluconate, 5 EGTA (glycol-bis(2-aminoethylether)-*N,N*′,*N*′,*N*′-tetraacetic acid), 10 Hepes (*N*-(2-hydroxyethyl)piperazine-*N*′-2-ethanesulfonic acid), 1 MgCl_2_, 2 Na_2_ATP, and 0.3 Na_2_GTP, pH 7.30. In experiments using slices from PLP-GFP mice, the following pipette solution was used (in mM): 130 KCl, 2 MgCl_2_, 0.5 CaCl_2_, 5 BAPTA (1,2-bis (*o*-aminophenoxy)ethane-*N,N,N,N*-tetraacetic acid), 10 Hepes, 3 Na_2_-ATP, pH 7.2. Patch pipettes were pulled from borosilicate glass capillaries (GB150(F)-8P, Science Products) using a horizontal puller (P-87, Sutter Instruments) and had a resistance of 3–7 MΩ. Patched astrocytes were clamped to -85 mV. In experiments using PLP-GFP mice, astrocytes and oligodendrocytes were clamped to -80 mV. Measurements were rejected if the series resistance exceeded 15 MΩ to ensure sufficient electrical and diffusional access to the patched cell (Pusch and Neher, 1988).

To generally determine the maturity of IC astrocytes we determined the *I–V* relationship of SR101-positive (SR101^+^) cells by applying a standard step protocol ranging from -150 to +50 mV with 10 mV increments and step duration of 50 ms. The resulting current traces were sampled at 30-50 kHz. Data were analyzed using “IGOR Pro” Software (WaveMetrics). After calculating the linear regression curve, two types of SR101^+^ cells could be distinguished according to their respective regression coefficient (see Kafitz et al., 2008): (1) non-passive astrocytes (*R*^2^ < 0.9983) and (2) passive astrocytes (*R*^2^ ≥ 0.9983).

### Tracer-Coupling

Gap junctional astrocytic and oligodendrocytic networks were visualized as described earlier (Langer et al., 2012; Griemsmann et al., 2015; Augustin et al., 2016). Astrocytes and oligodendrocytes were tracer- and dye-filled while patch-clamping for 20-130 min. In most experiments, intracellular solution contained a cocktail of the gap junction-permeable tracer neurobiotin (1%, Vector Laboratories, Inc.) and the gap junction-impermeable dye alexa fluor 568 (100 μM, Invitrogen). Neurobiotin was identified using avidin alexa fluor 488 (50 μg/ml, Invitrogen; Langer et al., 2012; Augustin et al., 2016).

In experiments using PLP-GFP mice, the intracellular solution contained biocytin (0.5%). Biocytin was labeled with streptavidin alexa fluor 647 (1:600, Molecular Probes). Simultaneously, PLP-GFP signal was immunohistochemically enhanced (Augustin et al., 2016). The primary antibody (chicken anti-GFP, ab13970, Abcam) was diluted 1:500 in 0.1% triton X-100 and 2% NGS (Millipore). The secondary antibody (goat anti-chicken alexa fluor 488, A-11039, Invitrogen) was diluted 1:500.

### Confocal Microscopy

Immunohistochemical labeling, SR101-labeling, and network tracing were documented at two confocal microscopes: (1) Zeiss LSM700 (EC Plan-Neofluar 10 ×/0.3; W N-Achroplan 40 ×/0.75 M27) in combination with ZEN software or (2) Leica TCS SP5 LSM (HC PL FLUOTAR 10× 0.30 DRY; HCX PL APO Lambda blue 63 × 1.4 OIL UV) using LAS AF software. Fluorophores were detected as described before (Augustin et al., 2016). To improve the quality of confocal micrographs and reduce background fluorescence, we used a Kalman filter (averaging of four identical image sections). In all experiments, a singly optical plane was documented. Images were processed using Fiji software (Schindelin et al., 2012).

**FIGURE 2:**
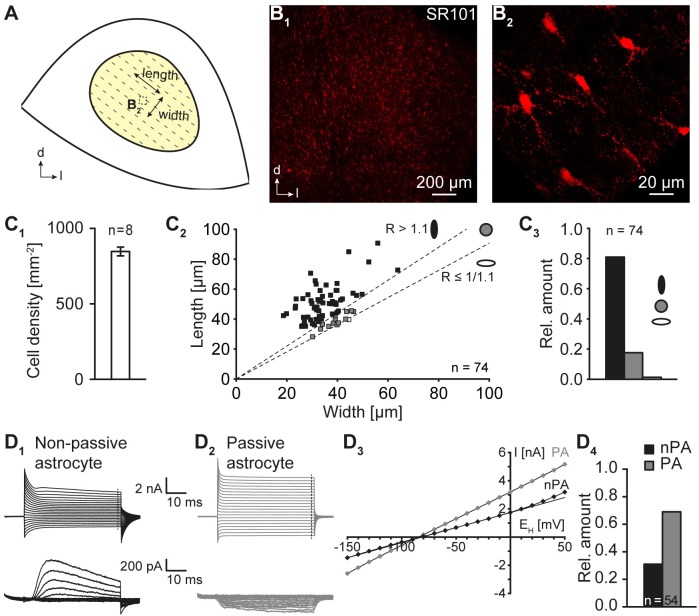
Morphological and electrophysiological properties of IC astrocytes. **(A)** Scheme depicting the central part of the inferior colliculus. The orientation of “length” and “width” is indicated. The dotted box denotes the location in which the astrocyte topography was subsequently analyzed. The tonotopic organization is indicated by dotted lines. **(B)** SR101-labeling of astrocytes in acute auditory brainstem slices. In the center of the IC strongly SR101-labeled astrocytes were found **(B_1_)** that exhibited a small soma with a diameter of about 10–15 μm **(B_2_)**. **(B_1,2_)** are independent from each other. **(C)** Astrocyte density and morphological properties. Astrocyte density was moderate. Numbers were corrected by 38% to allow the comparison with data from PFA fixed tissue (**C_1_**; see Section “Materials and Methods”). Processes of IC astrocytes were primarily oriented orthogonal to the tonotopic axis resulting in an elevated length/width ratio **(C_2_)**. Thus, most IC astrocytes exhibited an oval shape orthogonal to the tonotopic axis **(C_3_)**. Length: orthogonal to tonotopic axis. Width: longitudinal to tonotopic axis [see **(A)**]. **(D)** Electrophysiological properties of IC astrocytes. Astrocytes were voltage-clamped and step-wise hyper- and depolarized **(D_1,2_)**. Membrane currents were recorded before (top) and after isolation of voltage-activated outward currents (p/4, bottom). Non-passive astrocytes (nPA) expressed time- and voltage-dependent outward currents **(D_1_)**. Passive astrocytes (PA) lacked voltage-dependent outward currents **(D_2_)**. The *I-V* relationship was determined at the end of the voltage steps [dashed lines in **(D_1,2_)**]. Due to the presence of outward currents, nPAs exhibited a non-linear and PAs a linear *I-V* relationship (**D_3_**; see Section “Materials and Methods”). At P10-12, two third of IC astrocytes exhibited a non-linear *I-V* relationship **(D_4_)**. *n* represents the numbers of analyzed slices/cells and is provided within the diagram. Shown are mean values ± SEM.

In order to allow the comparison between astrocyte density and the density of tracer-labeled cells the shrinkage of acute tissue slices during PFA fixation after tracing experiments has to be considered. Accordingly, values of astrocyte density were corrected by 38% to match the “fixed” situation (Figure 2C_1_; Kafitz et al., 2008).

### Analysis of Gap Junctional Network

**FIGURE 3:**
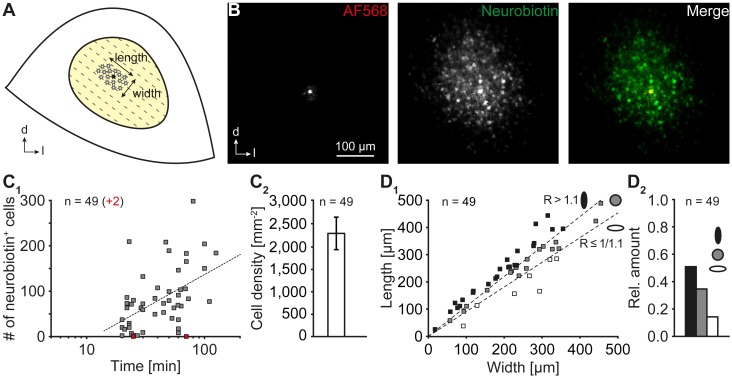
Astrocyte coupling. **(A)** Scheme depicting the central part of the inferior colliculus. The orientation of “length” and “width” is indicated. A schematic network is represented by asterisks. The patch-clamped cell is marked with a filled asterisk. The tonotopic organization is indicated by dotted lines. **(B)** The tracer neurobiotin diffused from a patch-clamped astrocyte (AF568) to neighboring cells. **(C)** Properties of IC networks. The network size depended on the duration of patch-clamping and concomitant injection of neurobiotin into an astrocyte **(C_1_)**. The increase of network size was slowed during prolonged tracer filling. Note: In two experiments, astrocytes lacked any coupling (red squares). The dotted line represents a logarithmic fit (*R*^2^ = 0.2342). IC networks exhibited a high cell density **(C_2_)**. The vast majority of networks had a non-spheroidal extension **(D_1_)** exhibiting an oval shape oriented orthogonally to the tonotopic axis **(D_2_)**. In **(D_1_)** one coordinate pair is located outside the dimensions of the diagram (width/length: 553/565). Length: orthogonal to tonotopic axis. Width: longitudinal to tonotopic axis [see **(A)**]. *n* represents the number of experiments and is provided within the diagrams. Shown are mean values ± SEM.

Patch-clamped astrocytes, which were initially filled with tracer, were identified via dialysis of their soma with alexa fluor 568. Tracer-labeled networks and astrocytes were classified into three groups depending on the ratio r, defined as the quotient of extension in direction orthogonal to tonotopic axis versus extension along tonotopic axis (see Figures 2, 3): (1) r > 1.1, oval shaped orthogonally to the tonotopic axis, (2) 0.91 (1/1.1) < r ≤ 1.1, spheroidal shaped, and (3) r ≤ 0.91 (1/1.1), oval shaped along the tonotopic axis (cf. Augustin et al., 2016). In order to analyze whether there is a preferred tracer-labeled network and astrocyte shape and orientation, we tested the normalized extension orthogonal to versus along the tonotopic axis. Data were normalized to values of extension along the tonotopic axis.

### Sodium Imaging

Cells in the IC were dye-loaded by bolus injection with the membrane-permeable form of SBFI (SBFI-AM; 500 μM; sodium-binding benzofurane isophthalate-acetoxymethyl ester; Invitrogen). Wide-field imaging was performed as described earlier (Langer and Rose, 2009; Langer et al., 2012) using a variable scan digital imaging system (TILL Photonics) attached to an upright microscope (BX51Wi, Olympus; 40 × water immersion objective, N.A. 0.8, Zeiss) and a CCD camera (TILL Imago VGA). Cells were alternately excited at 340 and 380 nm and fluorescence emission (>440 nm) was collected at 4 Hz from defined regions of interest (ROI) containing cellular structures of SR101^+^ cells and SR101-negative (SR101^-^) cells. SR101 fluorescence was excited at 565 nm and its emission was collected at >590 nm (Kafitz et al., 2008). After standard background subtraction of SBFI fluorescence (Langer and Rose, 2009), the fluorescence ratio (*F*_340_/*F*_380_) was calculated for individual ROIs and analyzed off-line by using OriginPro Software (OriginLab Corporation).

To analyze the intercellular spread of Na^+^ in cellular networks, single SR101^+^ cells were electrically stimulated (1 ms, 40 V) via an ACSF-filled microelectrode positioned on their cell body using an Isolated Pulse Stimulator (Model 2100, A-M Systems). This resulted in a large increase in the intracellular Na^+^ concentration of the stimulated cell as described earlier (Langer et al., 2012). Each cell was stimulated only once and experiments in which a rapid drop in the fluorescence emission at 340 nm was observed in response to the stimulation (indicative of cell damage) were discarded. Changes in intracellular Na^+^ concentrations in neighboring cells were normalized to the change in Na^+^ concentration evoked in the stimulated cell.

### Two-Photon Imaging

The correlation of SR101-labeling and expression of the PLP-GFP reporter was analyzed using two photon imaging as described before (Griemsmann et al., 2015; Augustin et al., 2016). Stacks of optical sections were obtained with a Leica TCS SP5 LSM, equipped with an infrared ultra-short-pulse laser (MaiTai; Spectra Physics). Two-photon absorption was achieved by excitation of the fluorophores with femtosecond pulses of infrared light with repetition rates of 80 MHz. The wavelength for dual excitation was adjusted for best signal to noise ratio for GFP and SR101 to 950 nm (0.5 W). Reflected light was collected with two channel non-descanned hybrid-detectors. Reflected emission light was separated with an FITC-TRITC filter cube (Leica). Image recording was performed with Leica LAS AF software.

Image analysis was performed using Fiji. Maximal mean somatic gray values of SR101 and PLP-GFP were obtained. Normalized gray value pairs were generated for each cell and plotted. The data indicated the presence of two distinct populations with either high or low intensity of PLP-GFP. Thus, we set manually a threshold and thereby generated two separate groups. These groups were utilized as starting points for a two dimensional cluster analysis based on a *k*-means algorithm (Matlab R2014b, The MathWorks; MacQueen, 1967). The components of value pairs were regarded as independent; therefore, the absolute difference between points (cityblock) was used for generating centroids (Bora and Gupta, 2014). These centroids served for the assignment of cells to one of the two groups. For validation of these new clusters, silhouette plots were generated, which illustrate the confidence of data point affiliation to a specific cluster (Rousseeuw, 1987).

### Statistics

**FIGURE 4:**
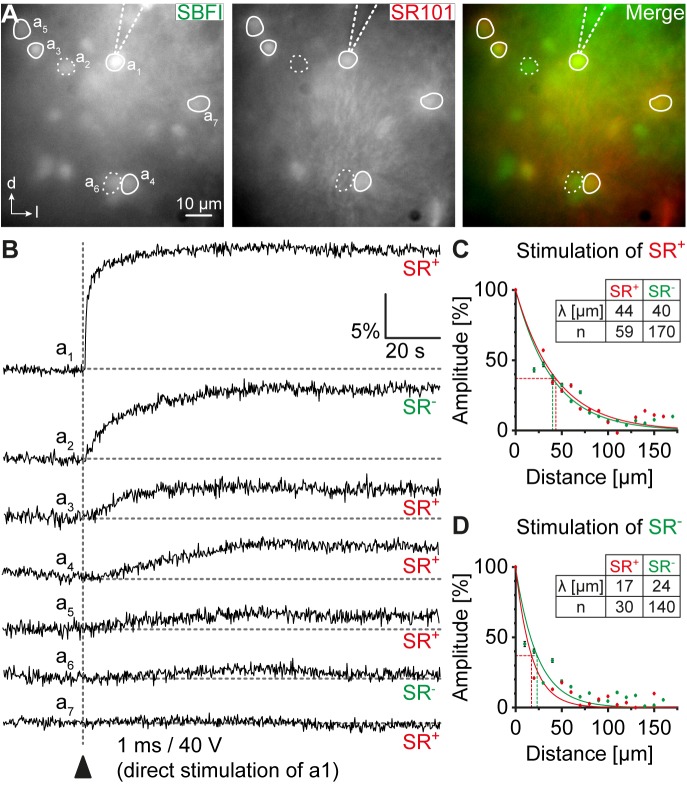
Na^+^ diffusion within glial networks. **(A)** IC cells were loaded with SBFI-AM (left). Astrocytes were identified by SR101-labeling (middle). **(B)** Electrical stimulation of a single astrocyte resulted in Na^+^ transients in the stimulated cell (a_1_) and in neighboring astrocytes (a_3_-a_5_, a_7_; SR^+^) and non-astrocytic cells (a2, a6; SR^-^). **(C,D)** Na^+^ diffusion in astrocytes and non-astrocytic cells could be elicited by stimulation of both astrocytes **(C)** and non-astrocytic cells **(D)**. The amplitude of Na^+^ transients depended on the distance from the stimulated cell. λ denotes the length constant of the decay. *n* represents the number of analyzed cells and is provided within the diagrams. Shown are mean values ± SEM.

Results were statistically analyzed using WinSTAT (R. Fitch Software). Data were tested for normal distribution with Kolmogorov-Smirnov test. In case of normal distribution, results were assessed by one-tailed, paired or non-paired Student’s *t*-tests. In the absence of a confirmed normal distribution, results were assessed by a Wilcoxon test for paired or an *U*-test (Mann–Whitney) for non-paired data. In experiments addressing Na^+^ transfer between glial cells in the IC, differences were tested by bin-wise comparison of mean amplitudes using a paired one-tailed Student’s *t*-test (Figures 4C,D). *p* represents the error probability, ^∗^*p* < 0.05, *p* < 0.01, ^∗∗∗^*p* < 0.001; *n* represents the number of experiments or cells/slices/animals. If not stated otherwise, data are provided as mean ± SEM.

## Results

### Expression of Cx43 and Cx30

Cx43 and Cx30 are expressed by IC astrocytes (Ball et al., 2007). To assess possible changes in their expression during early postnatal development, we analyzed Cx43 and Cx30 labeling pattern during the first 30 days after birth (Figure 1). One half of the fixed tissue slices was immunohistochemically processed for Cx43 and the second half for Cx30 as both Cx antibodies were raised in rabbit. We used the inwardly rectifying potassium channel (Kir) 4.1 for colabeling (cf. Augustin et al., 2016), which is moderately present in the IC (Moritz et al., 2015). Immunoreactivity for Cx43 was maximal at P12 and became less abundant during further development (*n* = 11-20/11-20/2; Figures 1B,C_1_). In contrast, Cx30 labeling was absent during early postnatal development and first appeared after P12 (*n* = 9-24/9-24/2; Figures 1B,C_2_). Kir4.1 levels mostly paralleled those of Cx43 (*n* = 22-37/22-37/2; Figures 1B,C_3_). Thus, our stainings indicate that at P10-13 (where subsequent tracing experiments were done), gap junctional coupling is mostly mediated by Cx43.

### Properties of IC Astrocytes

IC astrocytes were identified *a priori* by SR101-labeling (Stephan and Friauf, 2014; Ghirardini et al., 2018). IC astrocytes were homogeneously distributed within the central part of the IC (Figure 2B_1_) and their density amounted to 525 ± 18 cells/mm^2^ (*n* = 8/8/2). However, for later comparison with the density of tracer-filled cells it has to be considered that PFA fixation results in shrinkage of acute tissue slices causing an artificial increase in cell density. To compensate for this artifact, the density of IC astrocytes was corrected by 38% (Kafitz et al., 2008) resulting in 847 ± 29 cells/mm^2^ (*n* = 8/8/2; Figure 2C_1_).

IC astrocytes were characterized by a small soma with a diameter of about 10-15 μm and several fine processes with a length of up to 90 μm as detected by using confocal microscopy in acute tissue slices (Figures 2B_2_,C_2_). As LSO astrocytes exhibit a preferred orientation of their processes orthogonal to the tonotopic axis (Augustin et al., 2016), we next analyzed whether IC astrocytes are anisotropic as well. We found three classes of differently shaped IC astrocytes (Figure 2C_2_): (1) elongated astrocytes, whose processes were oriented predominantly orthogonal to the tonotopic axis (81%; 60 of 74 cells), (2) spheroidal astrocytes with radially equally distributed processes (18%; 13 of 74 cells), and (3) elongated astrocytes, whose processes were oriented predominantly along the tonotopic axis (1%; 1 of 74 cells). Thus, the majority of IC astrocytes showed an orientation orthogonal to the tonotopic axis (*n* = 74/6/2; *p* < 0.001; Figure 2C_3_).

Next, IC astrocytes were patch-clamped to determine their basic membrane properties. They exhibited a highly negative membrane potential (*E*_M_; -85.4 ± 0.8 mV, *n* = 54/54/44) and a low membrane resistance (*R*_M_; 10.1 ± 1.6 MΩ, *n* = 54/54/44) as reported earlier (Ghirardini et al., 2018). Both non-passive (31%, 17/54) and passive (69%, 37/54) astrocytes could be distinguished (Figure 2D; Kafitz et al., 2008), which is typical for astrocytes in the auditory brainstem during the second postnatal week (Augustin et al., 2016; Ghirardini et al., 2018).

### Glial IC Networks

To investigate gap junctional coupling in the IC, single astrocytes were patch-clamped and loaded with the gap junction-permeable tracer neurobiotin (Figure 3). Tracer-filled cells were visualized by avidin alexa fluor 488 (Figure 3B). IC astrocytes gave rise to large tracer-filled networks consisting of tens to hundreds of tracer-positive cells. As expected, the size of coupling networks depended on the loading time of the tracer (Figure 3C_1_). Coupling networks that were filled for at least 30 min—when increase of network size per time interval was less eminent—consisted of 100 ± 12 cells that occupied an area of 0.067 ± 0.009 mm^2^ (*n* = 32/32/31; not shown). Though large, the tracer-labeled networks never reached the borders of the central part of the IC. Notably, in 4% (2 of 51) of the experiments, the patch-clamped astrocyte was not tracer-coupled to other cells.

Furthermore, tracer-positive IC networks exhibited a relatively high cell density (2,275 ± 357 cells/mm^2^, *n* = 49/49/40; Figure 3C_2_) that exceeded the density of astrocytes in this region by a factor of 2.7 (“fixed”: 847 ± 29 cells/mm^2^, *n* = 8/8/2; Figure 2C_1_; *p* < 0.001). This indicates that SR101 labels only a subset of astrocytes and/or that coupling networks consisted of additional glial cells, e.g., oligodendrocytes, forming a panglial network.

IC astrocytes were anisotropic (Figure 2C_2,3_) and astrocyte anisotropy was shown to correlate with anisotropy of tracer-labeled networks (Anders et al., 2014; Augustin et al., 2016; Ghezali et al., 2018). Therefore, we next analyzed the topography of tracer-positive IC networks. We found three types of coupling networks (Figure 3D): (1) oval networks, which were oriented orthogonally to the tonotopic axis (51%, 25 of 49; see Figure 3A), (2) spheroidal networks (35%, 17 of 49), and (3) oval networks that were oriented along the tonotopic axis (14%, 7 of 49). Thus, most tracer-labeled networks were anisotropic and their orientation correlated predominantly with tonotopic organization (*n* = 49/49/40, *p* < 0.001).

### Intercellular Spread of Sodium in Glial Networks

To further characterize properties of IC networks, we studied the spread of Na^+^ between individual cells. To this end, SR101-labeled slices were additionally bolus-loaded with SBFI-AM. The Na^+^ indicator effectively labeled astrocytes as well as small-sized SR101^-^ cells (Figure 4A). In contrast, there was no obvious labeling of larger somata indicating that IC neurons did not efficiently take up SBFI (not shown). A small-diameter glass pipette was positioned in close vicinity of the cell body of a chosen astrocyte (see cell “a_1_” in Figure 4A). A brief current injection then resulted in an immediate rise of [Na^+^]_i_ in the directly stimulated cell (“a_1_”) due to its electroporation (Figure 4B; Langer et al., 2012; Augustin et al., 2016). In addition, neighboring cells showed a delayed increase in [Na^+^]_i_. These included both astrocytes and SR101^-^ cells (see cells “a_2_”–“a_7_”; Figure 4B), suggesting a panglial spread of Na^+^. The amplitude of Na^+^ transients induced by the electrical stimulation decayed mono-exponentially with increasing distance to the stimulated cell (SR101^+^: λ = 44 μm, *R*^2^ = 0.946; *n* = 59/9/4; SR101^-^: λ = 40 μm, *R*^2^ = 0.951; *n* = 170/9/4; Figure 4C), which is indicative of a diffusional spread of Na^+^ between gap junction-coupled cells (Langer et al., 2012; Augustin et al., 2016). No difference was found between SR101^+^ and SR101^-^ cells (bin-wise comparison of mean amplitude: *p* = 0.321).

When stimulating a SR101^-^ cell, the same phenomenon was observed: Na^+^ elevations were subsequently detected in both neighboring astrocytes and in SR101^-^ cells (Figure 4D). Again the amplitude of Na^+^ transients decayed mono-exponentially with increasing distance to the stimulated cell (SR101^+^: λ = 17 μm, *R*^2^ = 0.958; *n* = 30/4/2; SR101^-^: λ = 24 μm, *R*^2^ = 0.923; *n* = 140/4/2; Figure 4D). The homo-cellular Na^+^ transfer between SR101^-^ cells was slightly more effective than the hetero-cellular Na^+^ transfer between stimulated SR101^-^ cells and neighboring SR101^+^ cells (bin-wise comparison of mean amplitude: *p* = 0.021). Independent from the identity of the neighboring glial cell, Na^+^ transients exhibited stronger spatial restriction when directly stimulating SR101^-^ cells (bin wise comparison of mean amplitude: *p* = 0.002), indicating lower coupling efficiency of this population.

Taken together, these results show that Na^+^ can easily spread between coupled cells. The time course and decay kinetics of Na^+^ transients argue for a diffusion-mediated process. Na^+^ spread includes astrocytes as well as SR101^-^ cells further indicating the presence of functional panglial networks in the IC.

### Panglial IC Networks

**FIGURE 5:**
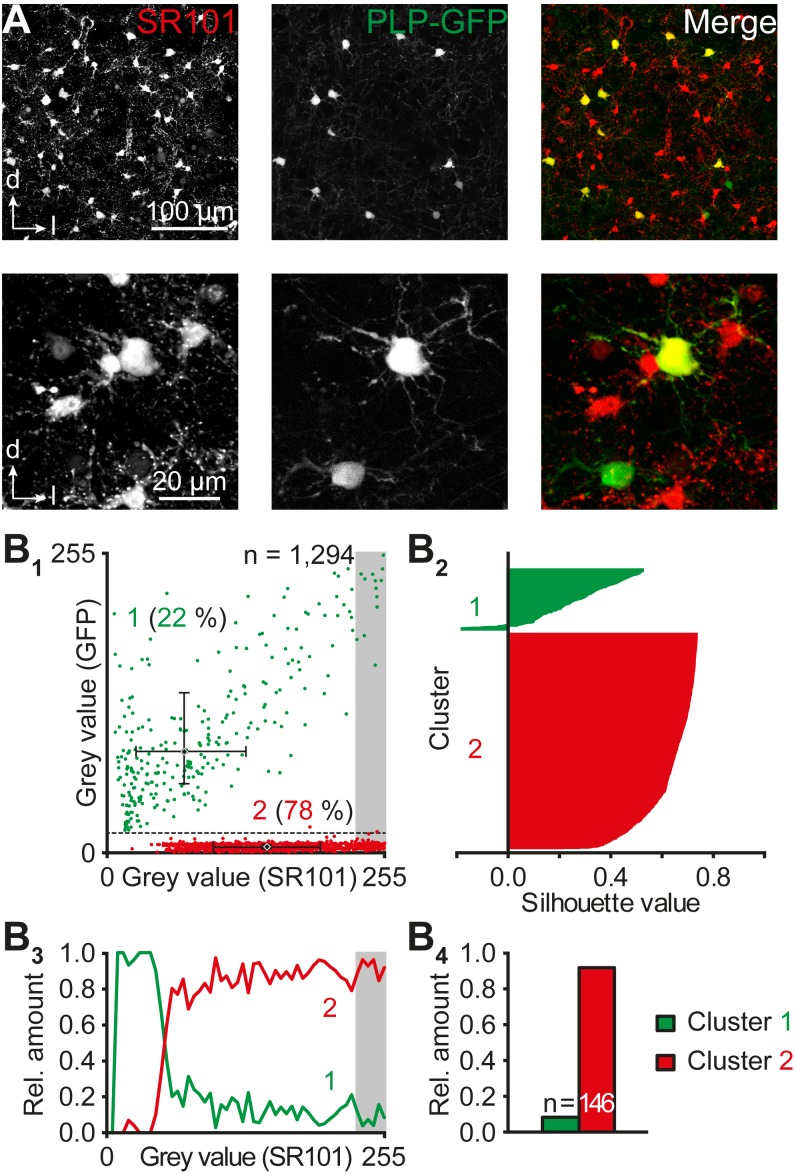
Astrocyte-oligodendrocyte proportion. **(A)** Heterogeneous levels of SR101 and PLP-GFP in cells of the central part of the IC. Upper panels: maximum projections of 10 consecutive optical slices with a step size of 1 μm. Lower panels: maximum projections of 80 consecutive optical slices with a step size of 0.25 μm each documented with higher magnification. **(B)** Population analysis of SR101^+^ and PLP-GFP^+^ cells in the IC. The scatter plot depicts somatic mean gray values of SR101^+^ and PLP-GFP^+^ cells **(B_1_)**. Each cell was colored depending on its *post hoc* allocation to a distinct group using cluster analysis. Centroids are depicted as diamonds and median values, 25% quartiles, and 75% quartiles for *x* and *y* direction of both clusters were depicted in the scatter plots **(B_1_)**. Data were illustrated in silhouette plots of *k* means-based cluster analysis (**B_2_**; see Section “Materials and Methods”). The relative cell proportion of both clusters was plotted against their SR101 fluorescence **(B_3_)**. The cells with strongest SR101-labeling (gray values: top 10% of the spectrum) were highlighted with a gray box **(B_1,3_)** and their relative amount was illustrated **(B_4_)**. *n* represents the number of cells reflecting the top 10% of the SR101-labeling spectrum and is provided within the bars.

Both the difference in the density of IC astrocytes and tracer-filled cells in IC networks as well as bi-directional Na^+^ signaling between astrocytes and non-astrocytic (SR101^-^) cells pointed toward the presence of panglial networks. Therefore, we assessed the possibility that oligodendrocytes contribute to the formation of panglial IC coupling networks using PLP-GFP reporter mice. Two-photon imaging revealed different classes of fluorescent cells: SR101^+^/GFP^-^ cells (astrocytes), GFP^+^ cells (oligodendrocytes) exhibiting weak to strong SR101 fluorescence (*n* = 1,294/4/4; Figure 5A). Two cell populations were separated by a scatter plot after manually setting a threshold (Figure 5B_1_): (1) PLP-GFP^+^ cells exhibiting a minimum PLP-GFP gray value >20/255 (22%, 280/1,294 cells: *x*/*y* coordinates of the population (median): 71/86, *n* = 280) and an overall weaker SR101 fluorescence, and (2) PLP-GFP^-^ cells with a PLP-GFP value ≤20/255 (78%, 1,014/1,294 cells; *x*/*y* coordinates of the population (median): 146/5, *n* = 1,014), which were SR101^+^.

Cluster analysis using *k* means verified our initial assumption of populations (Figure 5B_2_): cluster (1) SR101^+/-^/PLP-GFP^+^ cells morphologically resembling oligodendrocytes (*x*/*y* coordinates of centroid: 71/86) and cluster (2) SR101^+^/PLP-GFP^-^ cells with a typical morphology of astrocytes (*x*/*y* coordinates of centroid: 147/5). Only two out of 1,294 cells were differently affiliated compared to the manually set threshold (Figure 5B_1_). These two cells were likely astrocytes as their coordinates were closer to the second (astrocyte) cluster. The almost complete overlap between medians and centroids as well as the low error between our initial assumption and cluster analysis of 0.15% demonstrates the robustness of cell affiliation. The existence of a small group of SR101^+^/PLP-GFP^+^ cells raised the question regarding the reliability of SR101 as a marker for IC astrocytes. The relative proportion of astrocytes (cluster 2) increased with stronger SR101-labeling (92%, *n* = 146; gray value: top 10% of the SR101 spectrum; Figure 5B_3,4_). Accordingly, the relative amount of oligodendrocytes decreased to 8% in most brightly SR101-labeled cells.

**FIGURE 6:**
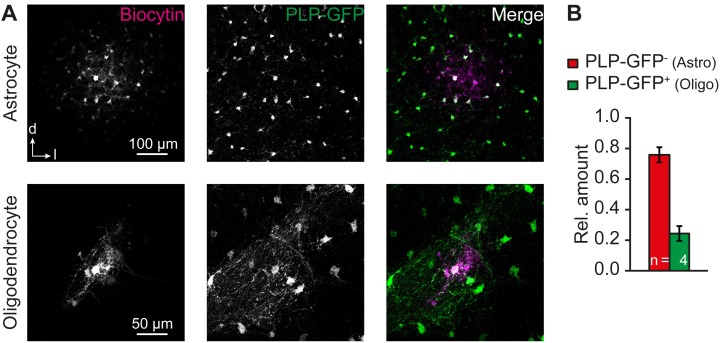
Astrocyte-oligodendrocyte coupling. **(A)** Panglial IC biocytin-labeled networks at P12–13. Biocytin diffused from patch-clamped astrocytes (upper row, two experiments) and oligodendrocytes (lower row, two experiments) into neighboring cells. **(B)** About 1/4 of the tracer-filled cells were PLP-GFP^+^ oligodendrocytes. PLP-GFP^-^ cells were assumed to be astrocytes. *n* represents the numbers of experiments and is provided within the bars. Shown are mean values ± SEM.

To further demonstrate the presence of panglial IC networks, we injected biocytin into IC astrocytes and IC oligodendrocytes from PLP-GFP mice. Tracer-filled networks contained astrocytes (PLP-GFP^-^) and oligodendrocytes (PLP-GFP^+^; Figure 6A). Notably, astrocyte filling produced larger tracer-coupled networks than respective filling of oligodendrocytes (A: 169 ± 39 cells, *n* = 2/2/2; O: 12 ± 2 cells, *n* = 2/2/2; not shown). This correlates with the observation of shorter length constants for Na^+^ redistribution when stimulating SR101^-^ cells instead of astrocytes (Figures 4C,D). For quantification of relative astrocyte and oligodendrocyte proportion in panglial IC networks, results were pooled as it can be assumed that the ratio is independent from the patched cell type (cf. Griemsmann et al., 2015). The A:O ratio was about 3:1 (76%/24%, *n* = 4/4/4), which is similar to the relative amount of astrocytes and oligodendrocytes in this nucleus (Figure 5B_1,2_). We conclude from these results that astrocytes and oligodendrocytes form panglial IC networks.

Taken together, our results show that tracer-filled glial networks in the IC (1) are mostly anisotropic correlating with anisotropic astrocyte topography, (2) consist of astrocytes and oligodendrocytes, and (3) allow the passage of ions (Na^+^), indicating functional coupling.

## Discussion

In the present study, we investigated gap junctional coupling in the center of the IC. Like the LSO, the IC exhibits tonotopic organization. Thus, we hypothesized that the anisotropy of tracer-filled networks seen in the LSO might also be present in the IC. Our data show that the gap-junction protein Cx43 is expressed throughout postnatal development, whereas Cx30 is only detectable after the second postnatal week. IC networks and IC astrocytes are anisotropic and oriented orthogonal to the tonotopic axis correlating with dendrite topography of IC neurons. In addition, IC networks are formed jointly by astrocytes and oligodendrocytes and are both able to redistribute Na^+^ within the gap junction network.

IC astrocytes are homogeneously distributed (Hafidi and Galifianakis, 2003) and were identified here *a priori* using SR101-labeling (Stephan and Friauf, 2014; Ghirardini et al., 2018). They exhibited a linear or outwardly rectifying *I–V* relationship (Figure 2) and—due to their generally high K^+^ permeability (Stephan et al., 2012)—a highly negative *E*_M_ and a low *R*_M_. This is typical for classical astrocytes in various auditory brainstem nuclei for that developmental stage (Muller et al., 2009; Reyes-Haro et al., 2010; Uwechue et al., 2012; Stephan and Friauf, 2014; Augustin et al., 2016; Ghirardini et al., 2018) and in general marks the developmental transition from immature to mature astrocytes (Schools et al., 2006; Kafitz et al., 2008).

### Formation of Networks

The formation of gap junction coupling depends on Cx expression. It was shown before that Cx43 and Cx30 are present in the IC of adult animals (Nagy et al., 1999; Ball et al., 2007; Kim et al., 2013). However, developmental expression was not analyzed so far. Here, we found an early expression of Cx43 that partially declines with age and a delayed developmental onset of Cx30 (Figure 1). Similar observations were made in hippocampus, thalamus, and LSO (Nagy et al., 1999; Griemsmann et al., 2015; Augustin et al., 2016). As we performed the tracing experiments at P10–13, Cx43 but not Cx30 is assumed to mediate astrocytic gap junctional coupling in this study. Tracer injection into single astrocytes gave rise to large numbers of labeled cells (Figure 3).

### Astrocyte and Network Anisotropy

IC astrocytes form large networks and like in other brain regions only rarely remain uncoupled (Figure 3; Houades et al., 2008; Augustin et al., 2016). IC astrocytes and IC tracer-labeled networks exhibited a predominantly oval shape that was oriented orthogonal to the tonotopic axis of the IC (Figures 2, 3). Anisotropic coupling networks are present in various brain regions. These result from (1) diffusion barriers, e.g., in the barrel cortex, thalamic barreloid fields, and olfactory glomeruli (Houades et al., 2008; Roux et al., 2011; Claus et al., 2018), or (2) anisotropy of tracer spreading, e.g., in the hippocampus, LSO or trigeminal nucleus (Anders et al., 2014; Augustin et al., 2016; Condamine et al., 2018). In some brain regions, a correlation between astrocyte anisotropy and coupling network anisotropy is observed (Anders et al., 2014; Augustin et al., 2016). Cx30 was reported to control astrocyte polarization and subsequently coupling network topography (Ghezali et al., 2018). However, it is rather unlikely that astrocyte and coupling network anisotropy in the IC was induced by Cx30 expression, because Cx30 is virtually absent in the IC at early postnatal stages (Figure 1). In the tonotopically organized LSO a similar anisotropy of both astrocytes and tracer-filled networks is present, which correlates with dendrite topography of principal neurons (Augustin et al., 2016). Likewise, astrocyte topography and anisotropy of tracer spread in the IC correlated with the dendrite organization of IC neurons (Oliver and Morest, 1984; Bal et al., 2002; Malmierca et al., 2011; Ghirardini et al., 2018). Therefore, we hypothesize that similar processes—though not unraveled yet—may lead to anisotropy in coupling efficiency in both LSO and IC.

Two basic questions arise: (1) Is the anisotropy in coupling efficiency beneficial in the auditory brainstem? We proposed before that it could be supportive for proper information processing in a tonotopically organized nucleus (Augustin et al., 2016) as ions and other signaling elements will be distributed rather within isofrequency bands than along the tonotopic axis. Thereby, putative cross talk between neighboring isofrequency bands could be limited, which might be in favor of precise tonotopic information processing. (2) Is this astrocyte and coupling anisotropy then a general feature of tonotopically organized auditory brainstem nuclei? Interestingly, in the medial nucleus of the trapezoid body (MNTB), another tonotopically structured nucleus (Kandler et al., 2009), astrocyte-derived tracer-filled networks are present, but were not explicitly reported to exhibit any specialized topography (Muller et al., 2009). However, the main synapse in the MNTB is the Calyx of Held synapse that directly engulfs the soma of a principle neuron (Borst and Soria van Hoeve, 2012). Aside this, MNTB principal neurons possess highly branched, short range dendrites that receive few inputs (Sommer et al., 1993; Smith et al., 1998). Thus, the topography of MNTB principal cells clearly contrasts with the narrow topography of LSO and IC principal cells, which possess long and mainly bipolar oriented dendrites (Oliver and Morest, 1984; Sanes et al., 1992a,b; Rietzel and Friauf, 1998; Bal et al., 2002; Malmierca et al., 2011; Ghirardini et al., 2018). It might be concluded from these studies that the topography of MNTB neurons is paralleled by astrocyte isotropy and isotropic coupling efficiency. Accordingly, astrocyte and coupling anisotropy might be a general feature of those auditory brainstem nuclei, whose principle cells exhibit a narrowed, bipolar dendrite topography. However, further analysis of MNTB networks is needed to provide firm evidence for these speculations.

### Functional Panglial Networks

IC coupling networks were described before and most coupled cells were reported to be immuno-positive for the calcium-binding protein S100β that is expressed mainly by astrocytes. Accordingly, the amount of oligodendrocytes contributing to IC coupling networks was assumed to be low (Ball et al., 2007). In contrast to this assumption, we found a substantial discrepancy between the relative numbers of astrocytes and coupled cells (Figures 2, 3) indicating that at least one other type of glial cell is present within the network. Furthermore, our Na^+^ imaging experiments showed that SR101^-^ cells bi-directionally communicated with neighboring astrocytes (Figure 4) suggesting Na^+^ diffusion between both cell types, which is again indicative of a panglial IC network. Indeed, we found a considerable amount of oligodendrocytes (Figure 5) and tracing experiments revealed an A:O ratio of 3:1 in IC networks (Figure 6). Still, adding 1/3 of oligodendrocytes (Figure 5) to the SR101-labeled astrocytes (Figure 2) is not sufficient to reach the cell density in tracer-filled networks (Figure 3), suggesting the involvement of an additional cell type.

As shown for the corpus callosum, panglial networks can also include NG2 glia (Maglione et al., 2010; Moshrefi-Ravasdjani et al., 2017). However, in many other brain regions, including the MNTB, NG2 glia do not form gap junction networks (Wallraff et al., 2004; Houades et al., 2008; Muller et al., 2009; Xu et al., 2014; Griemsmann et al., 2015). Currently, we cannot exclude a contribution of NG2 glia to IC networks, as some of them may also express S100β, which has been utilized as an astrocyte marker for tracer-filled IC networks before (Ball et al., 2007; Karram et al., 2008).

Moreover, the number of astrocytes in the central part of the IC might be underestimated here. SR101-labeling is heterogeneous across brain regions. Astrocytes in the ventrolateral medulla remain unlabeled, whereas LSO and IC astrocytes are moderately labeled and hippocampal astrocytes exhibit strong labeling (Kafitz et al., 2008; Schnell et al., 2012, 2015; Stephan and Friauf, 2014; Augustin et al., 2016; Ghirardini et al., 2018). Here, we found a continuum of weakly to brightly SR101-labeled astrocytes (Figure 5). Thus, a fraction of IC astrocytes might be overlooked using SR101-labeling in combination with confocal or wide field microscopy thereby leading to an underestimation of astrocyte density.

Na^+^ diffusion within gap junction networks is observed in various brain regions and is here found in the IC as well (Figure 4). The extent of the spatial spread of Na^+^ elevations emanating from IC astrocytes was comparable to that described in the LSO or the hippocampus (Langer et al., 2012; Augustin et al., 2016), but about twofold farther than in the corpus callosum (Moshrefi-Ravasdjani et al., 2017). Within the IC, however, stimulated astrocytes carried the Na^+^ signal over a longer distance compared to stimulated putative oligodendrocytes (SR101^-^ cells). A similar observation was made in the corpus callosum, in which oligodendrocytes carried the Na^+^ signal over a much shorter distance (Moshrefi-Ravasdjani et al., 2017). This argues for an overall lower coupling efficiency of IC oligodendrocytes, which could arise from different permeability of astrocytic and oligodendrocytic connexins (Bedner et al., 2006). In line with this, we found that the oligodendrocyte-derived tracer-filled networks were significantly smaller than respective astrocyte-derived tracer-filled networks. However, this contrasts with findings from the thalamus, where tracer-filled networks are not significantly different in the number of coupled cells suggesting a rather homogeneous coupling efficiency of astrocytes and oligodendrocytes in that brain region (Griemsmann et al., 2015).

In accordance to the proportion of astrocytes and oligodendrocytes a similar A:O ratio of about 3:1 was found in the tracer-filled networks (Figures 5, 6). The relative amount of astrocytes and oligodendrocytes in tracer-filled networks was independent from the initially tracer-injected cell type. Other brain regions, namely the LSO and the thalamus, exhibit a different A:O ratio of about 1:1 (Griemsmann et al., 2015; Augustin et al., 2016). There, however, the A:O ratio was independent from the tracer injected cell type, too.

Astrocytes and oligodendrocytes are coupled via heterotypic gap junctions (Cx43:Cx47, Cx30:Cx32; Giaume and Theis, 2010). However, Cx30 is not or only weakly expressed in the IC at the developmental stage when we investigated gap junctional coupling (Figure 1). Therefore, heterotypic A:O coupling is probably mainly mediated by Cx43:Cx47 pairs. The Na^+^ transfer was slightly better promoted between A:A and putative O:O (SR101^-^ cells) pairs than between A:O pairs. This might be due to different permeability, which has been observed between homotypic and heterotypic gap junctions (Weber et al., 2004; Rackauskas et al., 2007; Zhong et al., 2017). Taken together, in panglial IC networks astrocytes have a higher capability of ion redistribution compared to other glia.

## Conclusion

In summary, our results demonstrate that astrocytes form functional panglial networks with oligodendrocytes in the IC. These exhibit an anisotropic coupling efficiency, allowing preferred intercellular diffusion orthogonal to the tonotopic axis. The coupling anisotropy correlates with astrocyte as well as dendrite topography and might be beneficial for regulated ion and neurotransmitter homeostasis. The exact mechanism that leads to the formation of the panglial networks and the specific anisotropic arrangement of astrocytes, neurons, and tracer-filled glial networks in the IC is not resolved yet.

## Ethics Statement

This study was carried out in accordance with the recommendations of the German Animal Protection Law as well as the guidelines for the welfare of laboratory animals released by the European Community Council Directive.

## Author Contributions

JS, CS, and CR designed the experiments. JS, SW, VA, JL, RJ, CP, and DW performed the experiments and/or analyzed the data. JS wrote the manuscript. SW, JL, RJ, CR, and CS contributed to the writing of the manuscript.

## Conflict of Interest Statement

The authors declare that the research was conducted in the absence of any commercial or financial relationships that could be construed as a potential conflict of interest.
